# Phospholamban Is Downregulated by pVHL-Mediated Degradation through Oxidative Stress in Failing Heart

**DOI:** 10.3390/ijms18112232

**Published:** 2017-10-25

**Authors:** Shunichi Yokoe, Michio Asahi

**Affiliations:** Department of Pharmacology, Faculty of Medicine, Osaka Medical College, 2-7 Daigaku-machi, Takatsuki, Osaka 569-8686, Japan; yokoe@osaka-med.ac.jp

**Keywords:** cardiac function, phospholamban, calcium signaling, hypoxia, ubiquitination

## Abstract

The E3 ubiquitin ligase, von Hippel–Lindau (VHL), regulates protein expression by polyubiquitination. Although the protein VHL (pVHL) was reported to be involved in the heart function, the underlying mechanism is unclear. Here, we show that pVHL was upregulated in hearts from two types of genetically dilated cardiomyopathy (DCM) mice models. In comparison with the wild-type mouse, both DCM mice models showed a significant reduction in the expression of phospholamban (PLN), a potent inhibitor of sarco(endo)plasmic reticulum Ca^2+^-ATPase, and enhanced interaction between pVHL and PLN. To clarify whether pVHL is involved in PLN degradation in failing hearts, we used carbonylcyanide *m*-chlorophenylhydrazone (CCCP), a mitochondrial membrane potential (MMP)-lowering reagent, to mimic the heart failure condition in PLN-expressing HEK293 cells and found that CCCP treatment resulted in PLN degradation and increased interaction between PLN and pVHL. However, these effects were reversed with the addition of *N*-acetyl-l-cysteine. Furthermore, the co-transfection of *VHL* and PLN in HEK293 cells decreased PLN expression under oxidative stress, whereas knockdown of *VHL* increased PLN expression both under normal and oxidative stress conditions. Together, we propose that oxidative stress upregulates pVHL expression to induce PLN degradation in failing hearts.

## 1. Introduction

The ubiquitin/proteasome system (UPS) and autophagy/lysosome pathways are the major pathways for intracellular protein degradation and play important roles in protein quality control and various biological processes such as protein folding, signaling transduction, tumorigenesis, molecular trafficking, and clearance [1,2,3]. Cardiac protein quality is regulated by these systems after ubiquitination. A deficiency of these systems causes abnormal protein turnover or accumulation of misfolded proteins, presumably leading to cardiac diseases [4,5,6,7]. In some cases, E3 ubiquitin ligases such as HECT domain and ankyrin repeat containing E3 ubiquitin protein ligase 1 (HACE1), atrogin-1, and muscle RING-finger protein 1 (MuRF1) expressed in the heart are involved in the cardiac metabolism and function via autophagy-mediated or proteasome-mediated degradation [8,9,10,11,12,13,14]. The von Hippel–Lindau (VHL) protein (pVHL) is a component of an E3 ubiquitin ligase complex [15], which is known to regulate hypoxia-inducible factor-1α (HIF-1α) stability in normoxia environment by mediating its polyubiquitination [16,17]. The degradation of HIF-1α by pVHL is known to be regulated through prolyl hydroxylase domain (PHD) proteins [17,18,19]. In intestinal epithelial cells, indomethacin-induced cell damage was mediated by pVHL activation through the degradation of collagen I and HIF-1α [20]. The degradation of HIF-1α was regulated through PHDs, whereas collagen I degradation was regulated through pVHL expression level. Although pVHL is reported to be involved in the heart function by HIF-1α regulation through PHDs [21], the role of pVHL in UPS-mediated protein degradation in the heart is questionable. In the heart, phospholamban (PLN) plays an important role in the regulation of calcium flux across the sarcoplasmic reticulum (SR) by inhibiting the sarco(endo)plasmic reticulum Ca^2+^-ATPase (SERCA2a). The diminished SR Ca^2+^ cycling through inappropriate interactions between PLN and SERCA2a attenuates the progression to heart failure [22,23]. PLN has been reported to be ubiquitinated at Lys^3^ prior to the induction of its degradation [14,24]. Furthermore, it was shown that the ubiquitination-mediated degradation of PLN was triggered by its phosphorylation and inhibited by its interaction with SERCA2a [24]. The potential role of E3 ligase that targets PLN suggests that pVHL may also be involved in this degradation process. Here, we demonstrate for the first time that pVHL contributes to the ubiquitination of PLN, thereby inducing its degradation, under oxidative stress conditions in failing hearts (e.g., dilated cardiomyopathy (DCM)). The increase in pVHL-mediated degradation of PLN results in the decreased inhibitory effect of PLN on SERCA2a, possibly leading to impaired SR Ca^2+^ cycling. Moreover, the overexpression of *VHL* in HEK293 cells resulted in a decrease in the expression level of PLN under hydrogen peroxide (H_2_O_2_) stress condition, whereas the knockdown of *VHL* in HEK293 cells increased PLN expression level both under normal and H_2_O_2_ stress conditions. Taken together, our results suggest that pVHL may act as one of the major E3 ligases that ubiquitinate PLN to induce its degradation under oxidative stress conditions in failing hearts.

## 2. Results

### 2.1. Upregulation of pVHL Expression and Its Interaction with PLN in Heart Tissues in Two Types of DCM Mice Models, TgPLN^R9C^ and NHE1-Tg Mice

**Figure 1:**
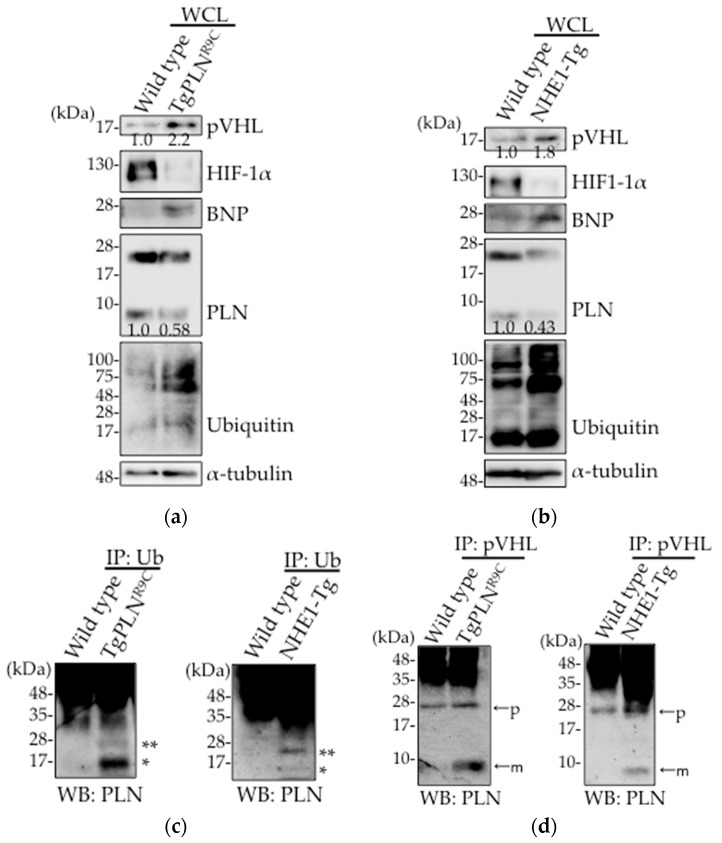
pVHL expression was increased and associated with PLN in hearts from TgPLN^R9C^ and NHE1-Tg mice. (**a**,**b**) Wild type (WT) and TgPLN^R9C^ (**a**) or NHE1-Tg mice (**b**) were sacrificed at 16 weeks of age and the hearts were homogenized. The lysates were subjected to Western blot analysis with antibodies against pVHL, HIF-1α, BNP, PLN, ubiquitin (Ub), and α-tubulin. The data designated indicate representative blots (*n* = 5); (**c**) The lysates from WT and TgPLN^R9C^ or NHE1-Tg mice hearts were subjected to co-immunoprecipitation with anti-Ub antibody, followed by Western blot analysis using anti-PLN antibody. * and ** indicate mono-ubiquitinated PLN (14.5 kDa) and di-ubiquitinated PLN (23 kDa), respectively; (**d**) The lysates from WT and TgPLN^R9C^ or NHE1-Tg mice hearts were subjected to co-immunoprecipitation with anti-pVHL antibody, followed by Western blot analysis using anti-PLN antibody. “p” and “m” indicate PLN pentamers and PLN monomers, respectively.

In intestinal epithelial cells, the expression level of pVHL is upregulated in response to oxidative stress induced by non-steroidal anti-inflammatory drugs (NSAIDs) [20]. We, therefore, examined pVHL expression level in heart tissues of both TgPLN^R9C^ (Figure 1a) and NHE1-Tg mice (Figure 1b). Although these mice are DCM models, their etiology are different from each other [25,26]. We used two different lines of mice to confirm the universality in failing hearts. We found that pVHL expression level was upregulated in the heart tissues from the two DCM mice types (Figure 1a,b). The levels of protein ubiquitination and brain natriuretic peptide (BNP) were both increased, indicative of the weak heart condition in both mice types. Decreased PLN expression was observed in these hearts, which is consistent with our previous observation [20]. Higher accumulation of mono- and di-ubiquitinated PLN was observed in both TgPLN^R9C^ and NHE1-Tg mice hearts (Figure 1c), as reported in HEK293 cells overexpressing PLN [24]. Oligo-ubiquitination of PLN is thought to trigger its poly-ubiquitination, thereby inducing degradation. As pVHL is known to play an important role in heart function by regulating HIF-1α through PHDs [21], we examined the expression level of HIF-1α. As shown in Figure 1a,b, HIF-1α was degraded in heart tissues from both transgenic mice, indicative of the upregulation of hydroxylation needed for HIF-1α degradation. To determine whether pVHL was involved in PLN ubiquitination, we examined the interaction between pVHL and PLN. A significant increase in pVHL–PLN interaction was observed in the pull-down fractions of pVHL from TgPLN^R9C^ and NHE1-Tg mice hearts as compared with controls (Figure 1d). Taken together, these data suggest that the upregulation in pVHL expression contributes to the degradation of PLN in DCM mice hearts.

### 2.2. pVHL Contributes to the Ubiquitination of PLN

**Figure 2:**
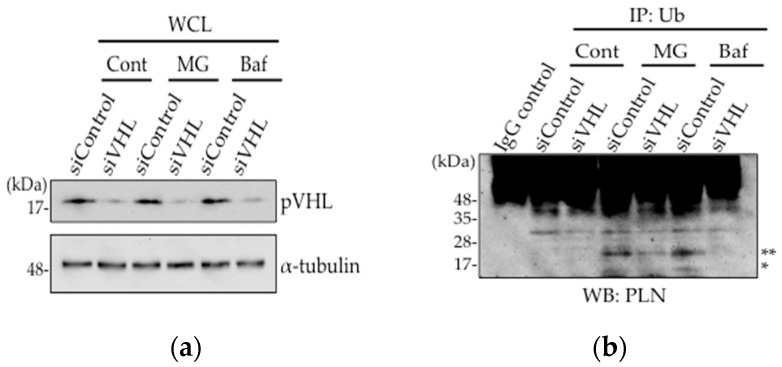
Ubiquitinated PLN was decreased after *VHL* knockdown. (**a**,**b**) *VHL* silencing using siRNA was performed in PLN-transfected HEK293 cells. Cells were treated with 10 μM MG132 or 100 nM bafilomycin for 3 h. Western blot analysis was performed using antibodies against pVHL and α-tubulin (**a**). Cell lysates were subjected to co-immunoprecipitation with anti-Ub antibody. After boiling to dissociate the non-covalent bonds, Western blot analysis were performed using anti-PLN antibody. * and ** indicate mono-ubiquitinated PLN (14.5 kDa) and di-ubiquitinated PLN (23 kDa), respectively (**b**).

As the expression level of pVHL and its interaction with PLN was increased in DCM mice hearts, we assessed whether pVHL was involved in the ubiquitination of PLN using *VHL*-silenced HEK293 cells (Figure 2a). Although mono-ubiquitination and di-ubiquitination of PLN were observed in PLN-transfected HEK293 cells upon treatment with, a proteasome inhibitor, 10 μM MG132 or a lysosome inhibitor, 100 nM bafilomycin, these ubiquitinations were dramatically decreased after 48 h of siVHL transfection, indicating that the ubiquitination of PLN by pVHL might be degraded in proteasome or lysosome (Figure 2b). Thus, pVHL is associated with the ubiquitination of PLN, thereby inducing its degradation.

### 2.3. Prevention of Carbonylcyanide m-Chlorophenylhydrazone (CCCP)-Induced PLN Degradation by N-acetyl-l-cysteine (NAC) Pretreatment in PLN-Transfected HEK293 Cells

**Figure 3:**
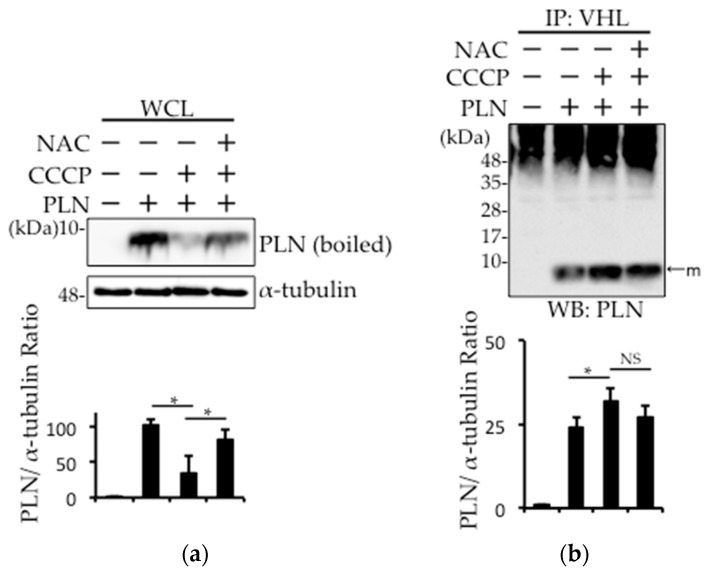

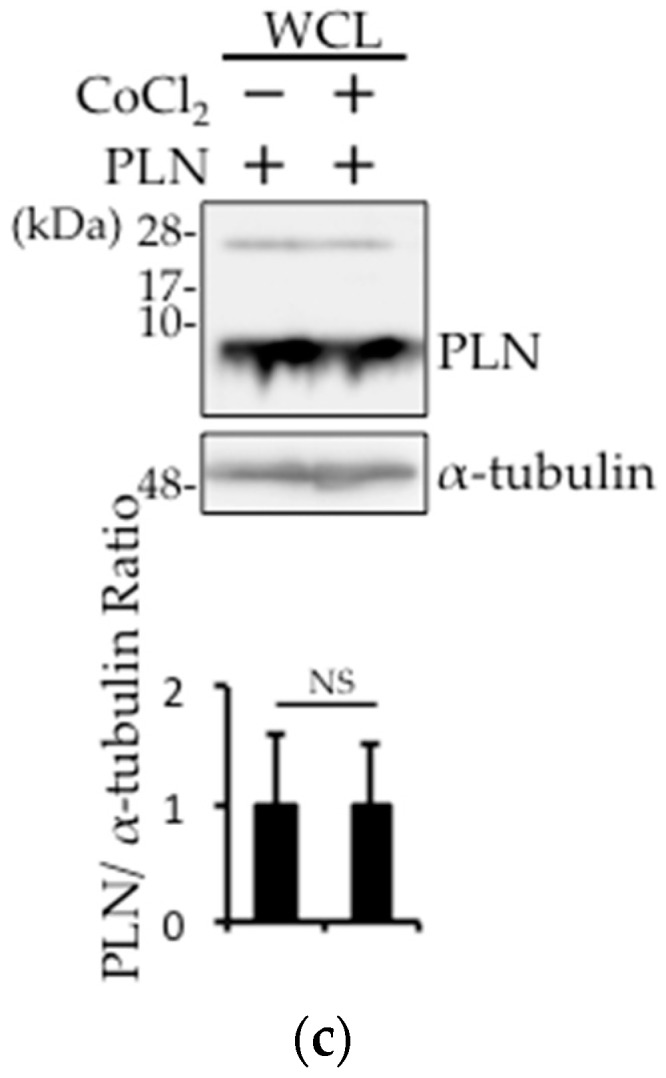
PLN was degraded by the disturbance in the mitochondrial membrane potential through an increase in the oxidative stress. (**a**,**b**) PLN-transfected HEK293 cells were harvested 4 h after treatment with 20 μM carbonylcyanide *m*-chlorophenylhydrazone (CCCP) with or without 5 mM *N*-acetyl-l-cysteine (NAC). Western blot analysis of whole cell lysates (**a**) and pVHL pull-down fractions (**b**) from PLN-transfected HEK293 cells was performed using an anti-PLN antibody. The bar graph indicates the intensity of PLN bands, as measured by ImageJ (Version 1.50i, National Institutes of Health, Bethesda, MD, USA) (*n* = 3). A value of *p* <0.05 (*) was considered significant. (**c**) PLN-transfected HEK293 cells were harvested 4 h after treatment with 125 μM CoCl_2_. Western blot analysis of whole cell lysates was performed using an anti-PLN antibody. The bar graph indicates the intensity of PLN bands, as measured by ImageJ (*n* = 3). NS: not significant.

In DCM, the damaged mitochondria generate superoxide radicals during cardiac remodeling. Therefore, we examined whether such radicals are involved in PLN degradation in PLN-transfected HEK293 cells. PLN expression was decreased after treatment with the mitochondria action potential uncoupler (CCCP), while *N*-acetyl-l-cysteine (NAC) pretreatment prevented this effect (Figure 3a). A cycloheximide chase assay was performed, and confirmed that the decreased PLN protein level in CCCP-treated cells is due to the degradation, not the reduction of synthesis (Figure S1). The pull-down fraction of pVHL from PLN-transfected HEK293 cells treated with CCCP showed that the interaction between pVHL and PLN was increased, while NAC treatment significantly reversed this effect (Figure 3b). To examine whether prolyl hydroxylase activity was required for pVHL–PLN interaction, we determined the effects of prolyl hydroxylase inhibitor, cobalt chloride (CoCl_2_), on PLN degradation in PLN-transfected HEK293 cells. As shown in Figure 3c, CoCl_2_ treatment had no effect on the expression of PLN.

### 2.4. The Involvement of pVHL in H_2_O_2_- or Isoproterenol-Mediated PLN Degradation

**Figure 4:**
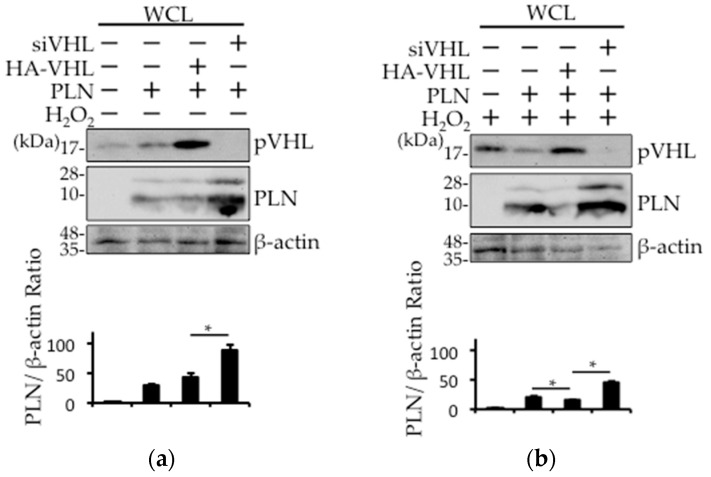

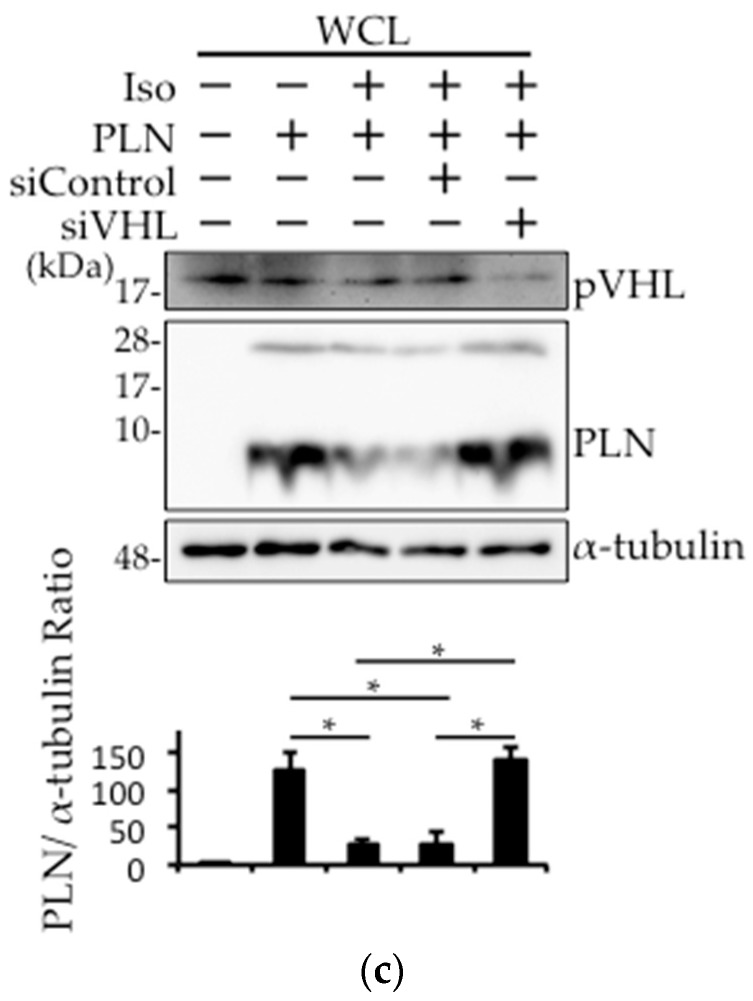
The expression of PLN is regulated by VHL. (**a**,**b**) *VHL* silencing or overexpression using siRNA or HA-VHL expression vector was performed in PLN-transfected HEK293 cells. The cells were harvested with (**b**) or without (**a**) 100 μM H_2_O_2_ treatment for 1 h. Western blot analysis was performed using antibodies against pVHL and PLN using whole cell lysates. The bar graph indicates the intensity of PLN bands, as measured by ImageJ (*n* = 3). A value of *p* <0.05 (*) was considered significant. (**c**) *VHL* silencing was performed in PLN-transfected HEK293 cells. The cells were harvested 1 h after treatment with 1 μM isoproterenol (Iso). Western blot analysis was performed using antibodies against pVHL and PLN using whole cell lysates. The bar graph indicates the intensity of PLN bands, as measured by ImageJ (*n* = 3). A value of *p* <0.05 (*) was considered significant.

Next, we examined whether pVHL was involved in isoproterenol- or H_2_O_2_-mediated PLN degradation. While the expression level of PLN was unchanged in the absence of H_2_O_2_, treatment with H_2_O_2_ resulted in a decrease in the expression level of PLN in hemagglutinin (HA)-tagged VHL (HA-VHL)–transfected HEK293 cells (Figure 4a,b). Furthermore, the expression level of PLN was increased with or without H_2_O_2_ treatment in siVHL-transfected HEK293 cells (Figure 4a,b). HA-mock vector (control) (Figure S2) or siControl (Figure 4c) did not change the VHL expression level. In addition, isoproterenol-mediated PLN degradation was prevented in siVHL-transfected HEK293 cells (Figure 4c). Isoproterenol-induced PLN reduction was not recovered with 50 ug/mL cycloheximide (CHX), an inhibitor of protein synthesis, indicating that the isoproterenol-induced PLN reduction may be due to the acceleration of PLN degradation (Figure S3).

## 3. Discussion

We have previously reported that the treatment of epithelial cells with NSAID increases oxidative stress, resulting in the upregulation in the expression of pVHL, thereby accelerating the intestinal cell damage [20]. As oxidative stress plays a major role in heart failure, we examined the expression level of pVHL in heart tissues of two types of DCM mice, TgPLN^R9C^ (Figure 1a) and NHE1-Tg (Figure 1b). As expected, pVHL expression was upregulated in heart tissues from both DCM mice. At the same time, the expression of PLN was significantly downregulated.

As PLN is a potent inhibitor of SERCA2a, the major regulator of cardiac function [23], the expression level and phosphorylation of PLN is critical for cardiac function. E3 ubiquitin ligases are expressed in different cell types and play an important role in cellular functions [27]. Studies have reported augmentation in the ubiquitination of cardiomyocytes in failing heart and its association with cardiac functions [28,29,30,31]. As shown in Figure 1c, higher accumulation of mono- and di-ubiquitinated PLN was observed in both TgPLN^R9C^ and NHE1-Tg mice hearts. These data indicate that PLN is one of the substrates modified by E3 ubiquitin ligases. As oligo-ubiquitination of PLN is thought to trigger its polyubiquitination and, consequently, degradation [24], PLN may be degraded through UPS in failing heart. To identify the E3 ubiquitin ligase responsible for PLN ubiquitination, we focused on pVHL that showed upregulated expression in failing hearts (Figure 1a,b). As a result, the interaction between pVHL and PLN was significantly increased in heart tissues from the two DCM mice types (Figure 1d). We silenced *VHL* in HEK293 cells and confirmed a decrease in the ubiquitination of PLN (Figure 2a,b). These results strongly indicate that VHL is one of the main E3-ubiquitin ligases that target PLN for degradation.

Oxidative stress, partly due to mitochondrial disturbance, is thought to be one of the factors that worsen heart failure [32,33]. Therefore, we examined the effects of a mitochondrial membrane uncoupler, CCCP, on PLN degradation in PLN-transfected HEK293 cells (Figure 3a,b). We found that CCCP induced PLN degradation, which was reversed in the presence of NAC, indicating that PLN was degraded partly by oxidative stress through mitochondrial disturbance.

The protein VHL is a tumor suppressor that forms E3 ubiquitin ligase complex, which negatively regulates HIF-1α by promoting its polyubiquitination [16,17]. It was reported that pVHL is involved in the stabilization of heart function via HIF-1α regulation [21]. The binding of pVHL to HIF-1α is regulated by the hydroxylation of specific prolyl residues at two functionally independent regions of HIF-1α [34]. To examine whether prolyl hydroxylase activity is required for pVHL–PLN interaction, we evaluated the effects of prolyl hydroxylase inhibitor (CoCl_2_) on PLN degradation in PLN-transfected HEK293 cells. As shown in Figure 3c, PLN expression level was unchanged after CoCl_2_ treatment, suggesting that hydroxylation plays no role in the interaction between the two proteins. Instead, PLN phosphorylation is thought to be indispensable for its interaction with pVHL [24], which results in the degradation of PLN by UPS.

To further confirm the pivotal role of pVHL in PLN degradation, we estimated the effects of *VHL* silencing and overexpression on pVHL and PLN expression. As shown in Figure 4a,b, PLN expression was downregulated in the presence of HA-VHL, but the treatment with siVHL resulted in the upregulation in the expression of PLN. It is interesting that the change in PLN expression was augmented in response to oxidative stress. Taken together, PLN undergoes degradation in the presence of upregulated pVHL expression in failing hearts in response to increased oxidative stress. Chronic ubiquitination of PLN by pVHL may exacerbate the cardiac function in DCM.

## 4. Materials and Methods

### 4.1. Materials

Carbonylcyanide *m*-chlorophenylhydrazone was purchased from Abcam (Cambridge, UK) and NAC was obtained from Sigma-Aldrich (St. Louis, MO, USA). Anti-pVHL antibody (Ab), anti-ubiquitin Ab, anti-BNP Ab, and anti-α-tubulin Ab were procured from BD Biosciences (San Jose, CA, USA), Santa Cruz Biotechnology (Dallas, TX, USA), Santa Cruz Biotechnology, and MBL (Nagoya, Japan), respectively. The targeting sequences of the siRNA against pVHL (siVHL) were as follows: sense, 5′-AGAAGGCCCUAAUGCUGGGTT-3′ and antisense, 5′-CCCAGCAUUAGGGCCUUCUTT-3′.

### 4.2. Animals

The TgPLN^R9C^ mice [25], transgenic mice with *PLN* point mutant R9C genetically overexpressed, were kindly provided by David H. MacLennan (University of Toronto, Toronto, ON, Canada). The NHE1-Tg mice [26], with sodium/hydrogen exchanger 1 (NHE1) genetically overexpressed, were a kind gift from Shigeo Wakabayashi (Osaka Medical College, Osaka, Japan). Both animals were used as severe DCM mice models. All animal procedures were performed according to the guidelines of the Osaka Medical College Animal Care and Use Committee (approval protocol number 27047; 31 March 2015).

### 4.3. Cell Culture and Plasmid DNA or siRNA Transfection

We maintained HEK293 cells in Dulbecco’s modified Eagle’s medium (DMEM) (Thermo Scientific, Waltham, MA, USA) supplemented with 10% fetal bovine serum, 100 U/mL penicillin, and 100 μg/mL streptomycin. Cultured HEK293 cells were transfected with PLN cDNA for 24 h, followed by their transfection with VHL-targeting small interfering RNA (siRNA) or HA-tagged VHL cDNA using TransIT-X2 reagent (Mirus, Madison, WI, USA), according to the manufacturer’s instruction.

### 4.4. Extraction of Mouse Heart Tissues

The mouse heart tissues were homogenized in a buffer (50 mM HEPES (pH 7.4), 5 mM sodium pyrophosphate, 10 mM sodium fluoride, 1 mM sodium orthovanadate, 10 mM β-glycerophosphate, and 1 mM phenylmethylsulfonyl fluoride) containing a protease inhibitor cocktail (ethylenediamine tetraacetic acid free) (WAKO Pure Chemical Industries, Osaka, Japan). The homogenates were centrifuged at 8000 rpm for 10 min and the supernatants were used for immunoprecipitation or Western blot analysis.

### 4.5. Western Blot Analysis

Mouse heart homogenates or HEK293 cell lysates transfected with PLN cDNA and siVHL or siControl were used for Western blot analysis. Following sodium dodecyl sulfate polyacrylamide gel electrophoresis (SDS-PAGE), the gel was placed into a semi-dry blotting system (Bio-Rad, Hercules, CA, USA), and the proteins were transferred onto a polyvinylidene fluoride (PVDF) membrane (Merck Millipore, Billerica, MA, USA). The membrane was blocked with 5% skimmed milk in Tris-buffered saline (TBS) containing 0.1% Tween-20 (TBST, blocking buffer), incubated with anti-PLN and -pVHL antibodies in blocking buffer, washed thrice with TBST, and then incubated with the secondary antibody conjugated with horseradish peroxidase. The membrane was visualized with Luminata Crescendo or Forte Western HRP substrate (Merck Millipore, Billerica, MA, USA), and the image was captured with ChemiDoc (Bio-Rad, Hercules, CA, USA).

### 4.6. Co-Immunoprecipitation

Co-immunoprecipitation was performed by using SureBeads Protein G Magnetic Beads (Bio-Rad, Hercules, CA, USA) according to the manufacturer’s instructions. The pull-down eluates were used for Western blot analysis with the antibodies of interest.

### 4.7. Statistical Analysis

The intensities of bands in the Western blot were quantified by ImageJ software (National Institutes of Health). The values are presented as the mean ± standard deviation (SD); *n* = 3 or more. Data were analyzed with the *F*-test for equality of variances, followed by the unpaired Student’s *t*-test for comparisons between two mean values. One-way analysis of variance with Tukey’s test was used for multiple comparisons. A value of *p* <0.05 was considered significant.

## 5. Conclusions

In conclusion, we propose that the increase in the oxidative stress results in the upregulation of pVHL expression and pVHL binding with PLN induces ubiquitination-mediated PLN degradation in failing hearts. PLN degradation through pVHL-mediated ubiquitination may act as one of the compensatory mechanisms to normalize cardiac function in failing hearts.

## Supplementary Materials

Supplementary materials can be found at www.mdpi.com/1422-0067/18/11/2232/s1.

## Abbreviations

SR	Sarcoplasmic reticulum
SERCA	Sarco(endo)plasmic reticulum Ca^2+^-ATPase
PLN	Phospholamban
WT	Wild-type
DCM	Dilated cardiomyopathy
CCCP	Carbonylcyanide m-chlorophenylhydrazone
NAC	*N*-acetyl-l-cysteine
UPS	Ubiquitin/proteasome system
VHL	Von Hippel–Lindau
HIF-1α	Hypoxia-inducible factor-1α
HACE1	HECT domain and ankyrin repeat containing E3 ubiquitin protein ligase 1
BNP	Brain natriuretic peptide
WCL	Whole cell lysate
WB	Western blotting
