# Supplementary material for: Phospholamban Is Downregulated by pVHL-Mediated Degradation through Oxidative Stress in Failing Heart
Source: Int J Mol Sci. 2017 Oct 25;18(11):2232. doi: 10.3390/ijms18112232 (PMC5713202; doi:10.3390/ijms18112232)
Supplement: Supplementary file 1 [file ijms-18-02232-s001.pdf]

International Journal of *Molecular Sciences*

Phospholamban Is Downregulated by pVHL-Mediated Degradation through Oxidative Stress in Failing Heart

Shunichi Yokoe and Michio Asahi *

Supplementary Materials

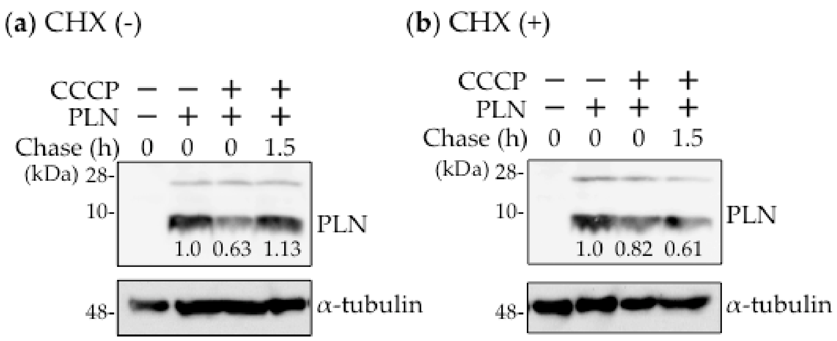

Supplementary Figure S1. (**a**,**b**) 20 μM carbonylcyanide m-chlorophenylhydrazone (CCCP)-treated HEK293 cells were washed to remove CCCP away, and chased for 1.5 h with (**b**) or without (**a**) 50 μg/mL cycloheximide (CHX).

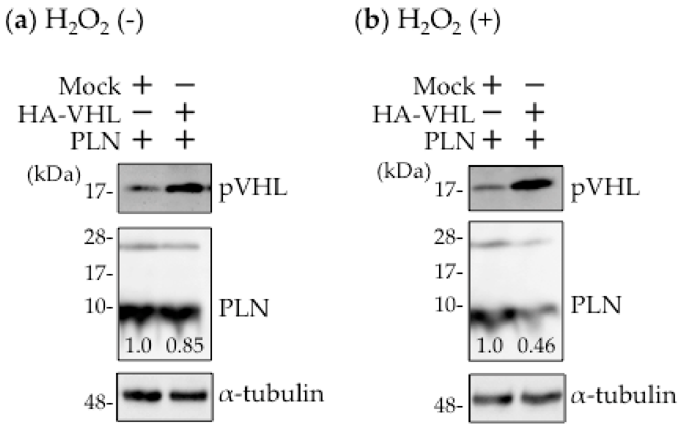

Supplementary Figure S2. (**a**,**b**) VHL overexpression using HA-VHL expression vector was performed in PLN-transfected HEK293 cells, compared with HA-mock overexpressed cells. The cells were harvested with (**b**) or without (**a**) 100 μM H_2O_2 treatment for 1 h.

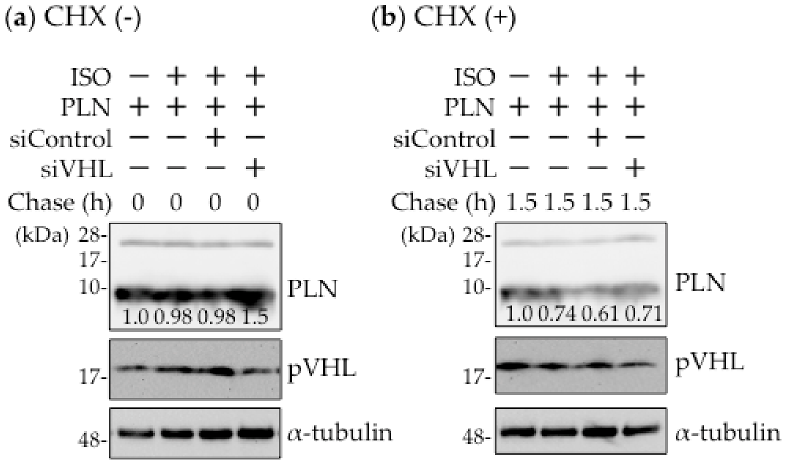

Supplementary Figure S3. (**a**,**b**) *VHL* silencing was performed in PLN-transfected HEK293 cells, compared with negative siRNA control (siControl). 1 μM isoproterenol (ISO)-treated HEK293 cells were washed to remove ISO away, and chased for 1.5 h with (**b**) or without (**a**) 50 μg/mL cycloheximide (CHX).
